# Comparison of Anesthetic Features in Diazepam and Midazolam for Sedation Dentistry: A Scoping Review

**DOI:** 10.7759/cureus.79079

**Published:** 2025-02-16

**Authors:** Takutoshi Inoue, Toru Yamamoto, Naotaka Kishimoto, Kenji Seo

**Affiliations:** 1 Department of Anatomy, Teikyo University School of Medicine, Tokyo, JPN; 2 Division of Dental Anesthesiology, Faculty of Dentistry and Graduate School of Medical and Dental Sciences, Niigata University, Niigata, JPN

**Keywords:** dental treatment, diazepam, intravenous sedation, midazolam, oral surgery, sedation dentistry

## Abstract

In recent years, there have been incidences of shortages of sedatives due to infectious diseases and supply issues. Dental anesthesiologists are required to have backup plans in case of supply shortages. Therefore, the aim of this study was to highlight the characteristics of diazepam and compare them with those of midazolam, and to explore the possibility of proposing useful situations for the use of diazepam in the modern intravenous sedation (IVS) scene. The study followed the Preferred Reporting Items for Systematic Reviews and Meta-Analyses Extension for Scoping Reviews (PRISMA-ScR) guidelines. Literature research was performed using PubMed and Google Scholar. After a detailed scrutiny, 20 English-language studies comparing midazolam and diazepam met the eligibility criteria. The evaluation points were classified into four categories: onset of action, recovery from sedation, injection pain, and amnesic effects. The results of this analysis review highlight that diazepam tends to have (a) a slower onset of action and recovery, (b) more injection pain, and (c) a weaker amnesic effect compared with midazolam, and that diazepam has to be administered at higher doses compared with midazolam. The use of two drugs in combination (e.g., midazolam + propofol) is now a common practice due to the advances in anesthetic drugs. In addition, dentistry has become more advanced and involves lengthy procedures, such as implant surgeries. Therefore, it is necessary to focus on the optimal drug dosages in the combination of diazepam and propofol, the postoperative recovery time, and the presence or absence of injection pain and amnesic effects. Diazepam may be an alternative to midazolam for IVS during prolonged dental procedures in situations of shortage and requires further study.

## Introduction and background

Dentists worldwide practice intravenous sedation (IVS) to reduce anxiety in patients and facilitate dental treatment [1]. Midazolam and propofol are commonly used for this purpose in Japan because of their short pharmacologic half-life and good adjustability [1]. However, in recent years, similar to other countries, Japan has experienced frequent shortages of midazolam (parenteral) and propofol because of the coronavirus pandemic (COVID-19) and other supply issues [2,3]. Drug shortages are a global problem and pose significant challenges to healthcare systems [4]. Therefore, dental anesthesiologists must be prepared for possible shortages of sedatives with economically efficient, safe, and convenient alternatives.

Midazolam is a benzodiazepine that exhibits sedative, anxiolytic, hypnotic, and amnesic properties and also has a short half-life [5]. Diazepam, flunitrazepam, and remimazolam are some other benzodiazepine options if midazolam is also in short supply in Japan [6,7]. Of these, flunitrazepam is not preferred for day-case anesthesia, since it has a significantly longer duration of action compared with midazolam and diazepam [8]. Remimazolam, an ultra-short-acting benzodiazepine, is associated with a rapid onset of action and recovery and is considered safe and effective in Japan for dental patients requiring IVS [7]. However, in Japan, it is only suitable for use in general anesthesia [7] and is expensive, thereby limiting its use as an alternative to midazolam.

Diazepam is a benzodiazepine that appeared before midazolam, and propofol appeared after both diazepam and midazolam. As a result, the controllability of IVS is improved compared with nitrous oxide inhalation sedation. Diazepam exerts an amnesic effect similar to midazolam but is associated with the limitations of injection pain (vascular pain) and a longer duration of action compared with midazolam [8], thereby limiting its preference among dental anesthesiologists in recent years. However, it is necessary to summarize the characteristics of diazepam from the perspective of modern anesthesiology in scenarios of drug shortages.

This study aimed to highlight the characteristics of diazepam in comparison with midazolam through a comprehensive literature review and to explore possible useful situations for the application of diazepam in dental clinics for IVS. This can provide valuable information for dentists practicing IVS and help identify the current research gaps in this area.

## Review

Methods

This scoping review aimed to identify prognostic factors for cervical cancer in Asian countries. The review followed the Arksey and O'Malley framework, comprising six systematic steps: identifying the research question; identifying relevant studies; study selection; charting the data; collating, summarizing, and reporting results; and consulting results [9].

Study design

This scoping review aimed to investigate and compare diazepam and midazolam. Our primary research questions were as follows: "How does diazepam compare with midazolam in terms of onset of action, recovery from sedation, injection pain (vascular pain), and amnesic effects (anterograde amnesia)?"

Search strategy

The study protocol was registered in the Open Science Framework (Registration DOI: 10.17605/OSF.IO/NXVDM) and was conducted in accordance with the Preferred Reporting Items for Systematic Reviews and Meta-Analyses Extension for Scoping Reviews (PRISMA-ScR) guidelines. PubMed and Google Scholar were used to search for English-language articles available online and published up to August 2024. The MeSH terms used for searching were: (midazolam) AND (diazepam) AND ((dental) OR (dental comparison)). References were managed using EndNote Basic (Clarivate).

Study selection

This review was conducted by authors from two different institutions to reflect the synthesis of opinions from different institutions and dental anesthesiologists. All researchers discussed the results and inclusion criteria to set a consistent base before commencing the review of the articles from the initial search. Study selection involved two steps: an initial screening (a title and abstract review) and an eligibility screening (a full-text review). Two researchers (T.I. and T.Y.) independently performed the two screenings, and in situations of discrepancies, the opinion of a third co-investigator (N.K.) was considered. After data acquisition, the research team discussed and drew conclusions under the supervision of the co-author (K.S.).

Inclusion and exclusion criteria

The criteria for the inclusion of full-text, peer-reviewed clinical articles comparing diazepam and midazolam were as follows: subjects aged 14 years or older; double-blind, crossover, prospective cohort studies; and prospective, randomized, double-blind, multicenter studies. Articles were excluded if they met any of the following criteria: animal studies, study protocols, reviews, guidelines, conference abstracts, case reports, or studies with inconsistent inclusion criteria. The criteria for comparison of the two drugs included: "onset of action," "recovery from sedation," "injection pain (vascular pain)," and "amnesic effects (anterograde amnesia)."

The articles that met the eligibility criteria were reviewed carefully and classified into four categories: onset of action, recovery from sedation, injection pain, and amnesic effect. Information on dosage and age was included in several articles; therefore, this study investigated the relationship between the four categories mentioned above. Since the dosage units (mg or mg/kg) were different in different studies, the dosage ratio (diazepam:midazolam ratio, or D:M ratio) was calculated and subsequently converted into a percentage. For example, D:M = 1:1 indicated 50% of each diazepam and midazolam. This enabled us to determine which drug was administered at a higher dose. The age, mean, maximum, and minimum values were examined to determine the strata that were studied.

Results

**Figure 1:**
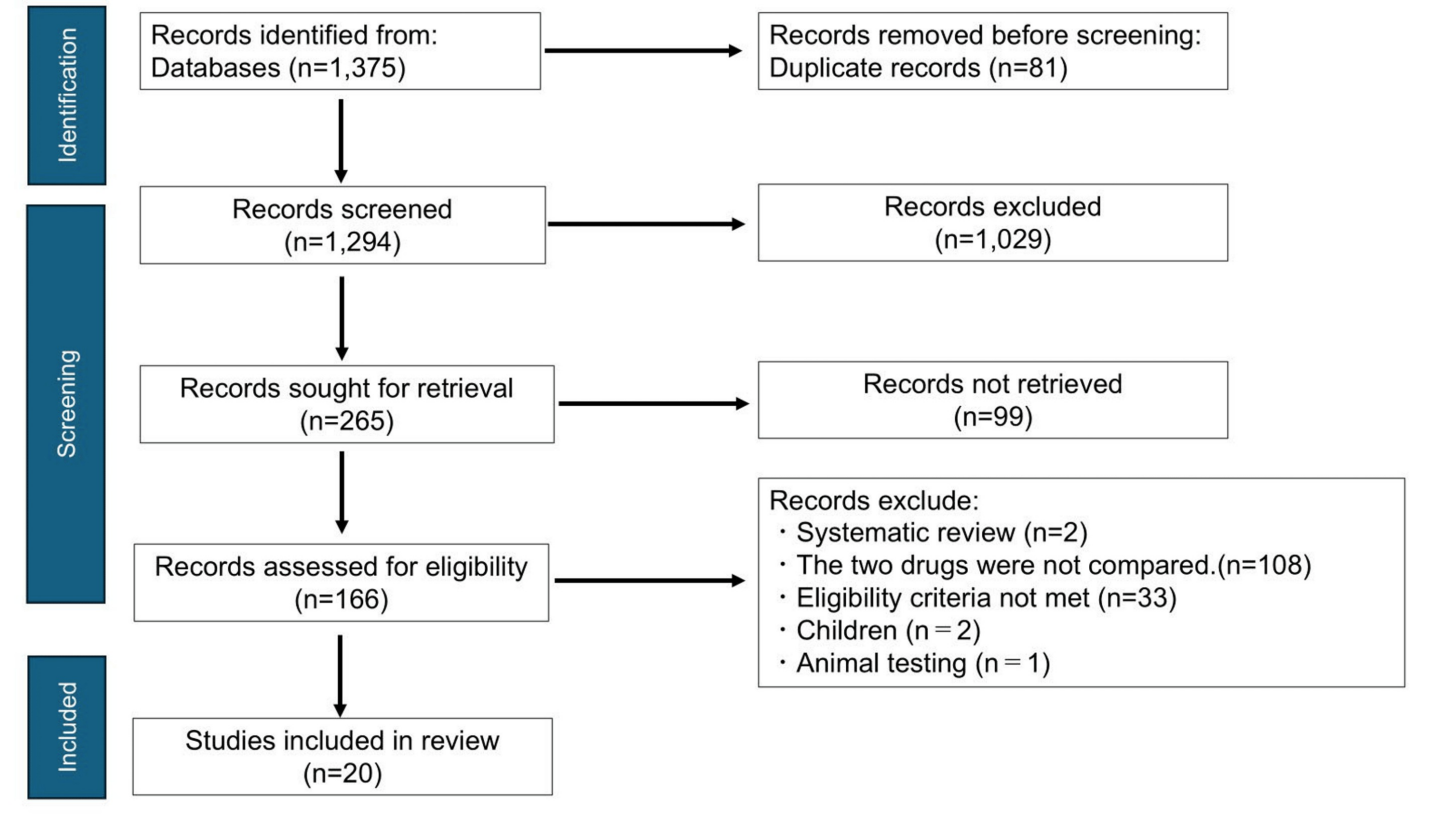
Identification of studies via databases with PRISMA flow diagram PRISMA: Preferred Reporting Items for Systematic Reviews and Meta-Analyses

Figure 1 depicts the methodological flow of this study. The search expression developed by the researchers yielded 1,375 results. Full-text articles were available for 166 studies. Finally, 20 articles met the eligibility criteria, and 146 articles were excluded.

**Figure 2:**
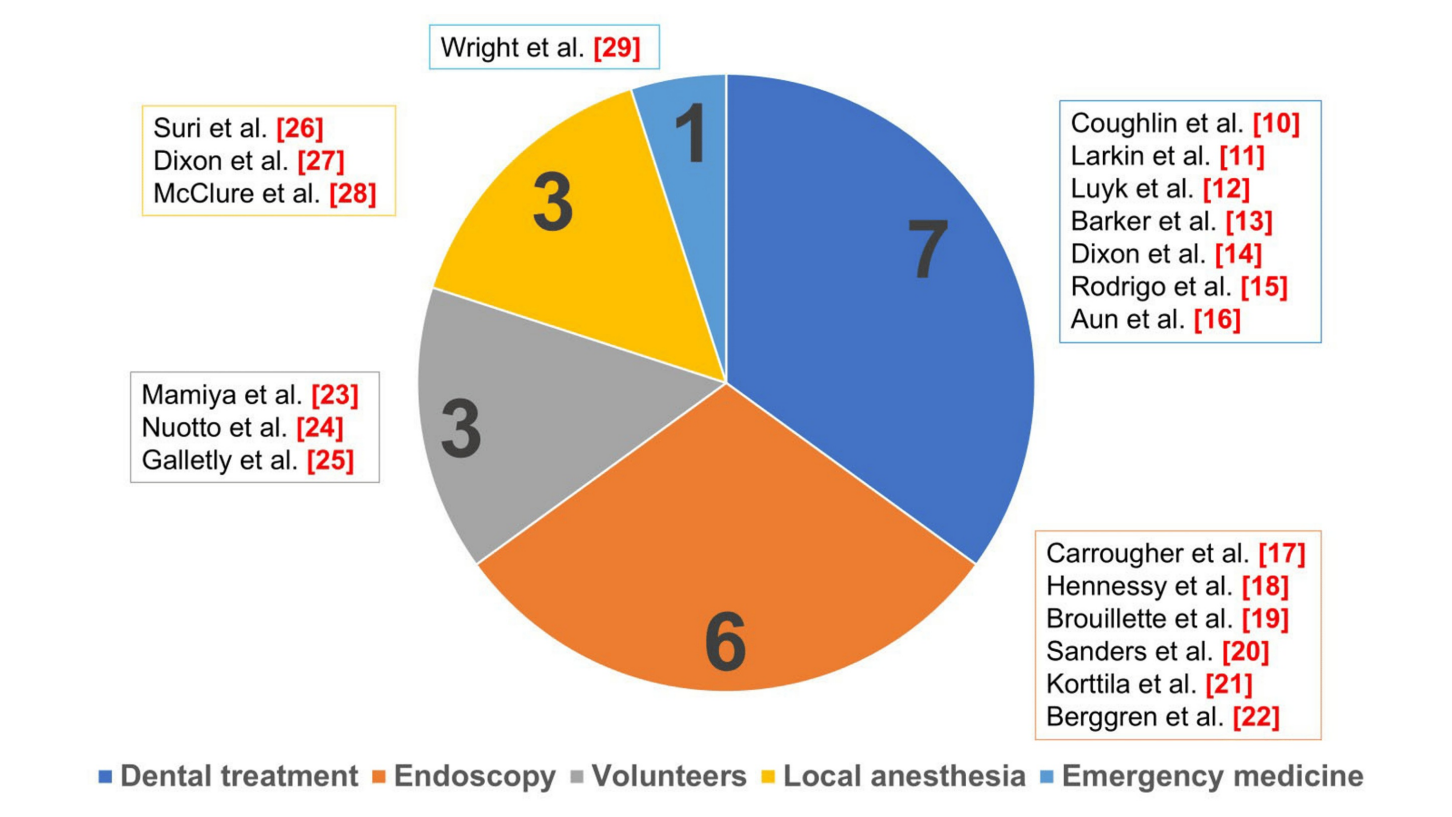
Fields of articles that met the eligibility criteria The 20 studies that met the eligibility criteria were subdivided into five areas.

The 20 studies that met the eligibility criteria included studies pertaining to the medical field, which were then subdivided based on the specialty (Figure 2). There were seven studies on dental treatment [10-16], six studies on endoscopy [17-22], three studies on volunteers [23-25], three studies on local anesthesia [26-28], and one study on emergency medicine [29].

Onset of action

The differences in the onset of action between diazepam and midazolam were reported by seven studies. Four studies reported diazepam to have a slower onset of action compared with midazolam [13,15,16,29], whereas three studies reported that the onset of action of the two drugs did not differ significantly or that there was no difference at all [24,25,27]. Thus, the fact that the onset of action of diazepam was slower compared with midazolam was supported by more evidence.

**Figure 3:**
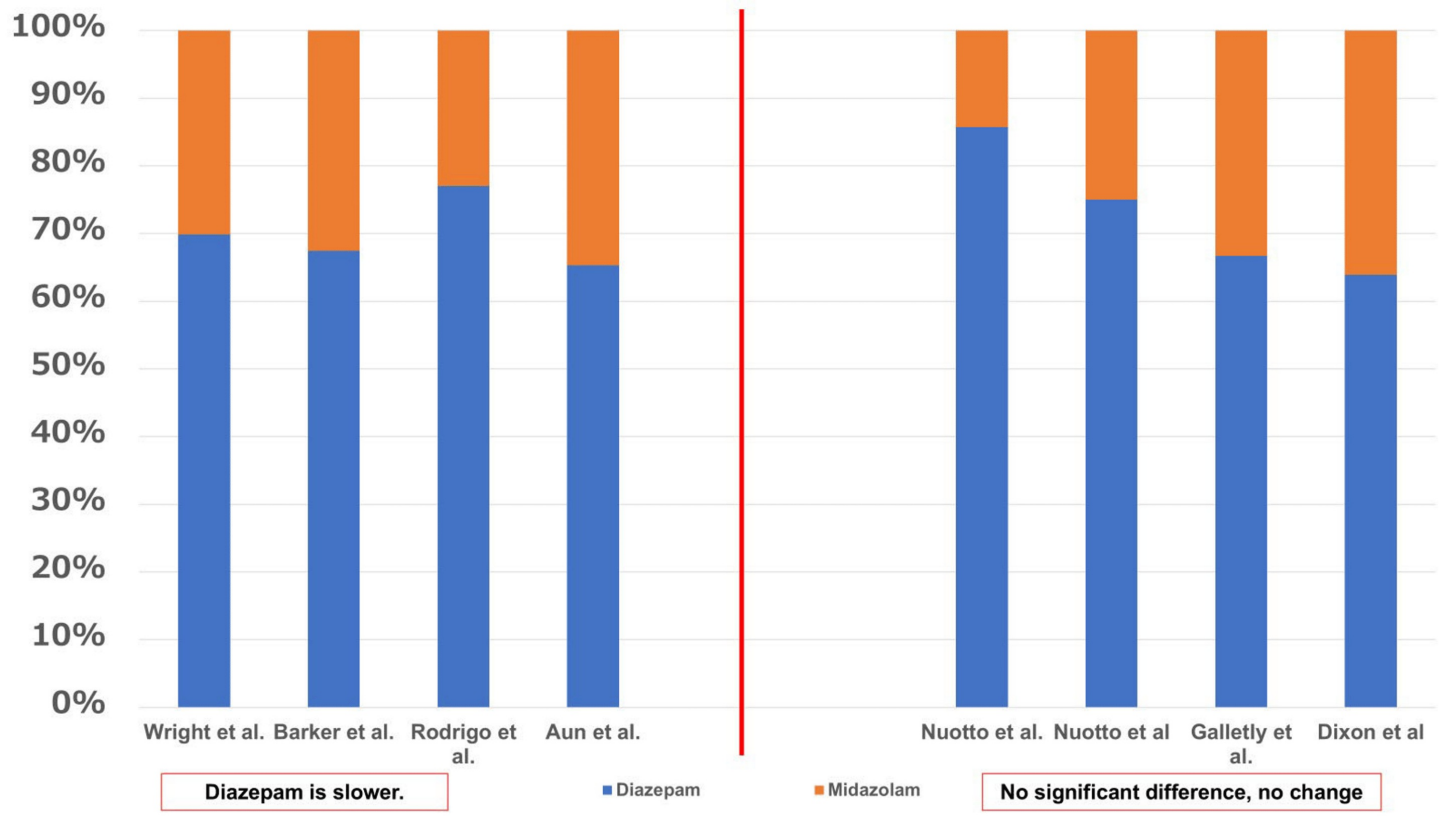
Dosage and onset of action From the results of seven studies [13,15,16,24,25,27,29], the dosage was examined. Because there was variation in the units (mg or mg/kg), they were converted to percentages. For example, 1 mg diazepam and 1 mg midazolam are 50% diazepam and 50% midazolam.

**Figure 4:**
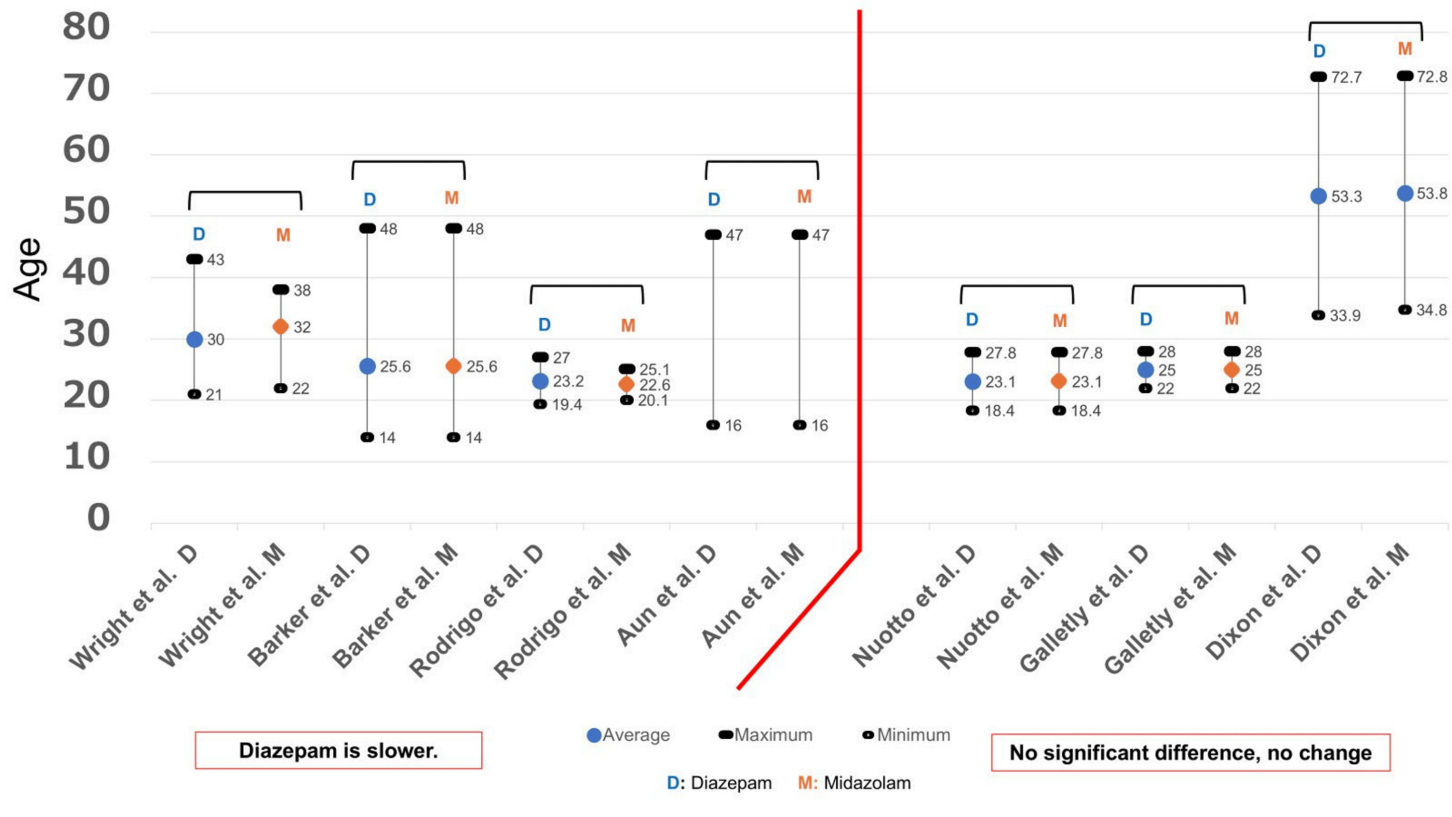
Age and onset of action From the results of seven studies [13,15,16,24,25,27,29], the age was examined. The maximum, minimum, and average values for each study are shown in this figure. There was one report that did not state the average age. D: Diazepam; M: Midazolam

The dosages were examined in seven studies (Figure 3). Studies conducted by Wright et al. [29], Barker et al. [13], Rodrigo and Clark [15], Aun et al. [16], Nuotto et al. [24], and Dixon et al. [27] examined the onset of action of diazepam and midazolam at their respective mean doses (mg or mg/kg). Galletly et al. [25] examined the onset of action of diazepam and midazolam at fixed doses of 10 mg and 5 mg in volunteers. The results have been shown in Figure 3. Nuotto et al. [24] reported that 0.3 mg/kg diazepam was equivalent to 0.05-0.1 mg/kg midazolam in volunteers, and the two results have been shown in Figure 4. According to the studies, the dose ratios (D:M ratio) demonstrating a slower onset of action for diazepam compared with midazolam ranged from 1.88:1 to 3.35:1, whereas reports showing no significant difference or no change in onset ranged from 1.77:1 to 6:1. Thus, with regard to the onset of action, all reports were unanimous in indicating that the dosage of diazepam was higher compared with that of midazolam.

The investigation conducted by seven studies was centered around the age of patients (Figure 4). Almost all studies provided the mean, maximum, and minimum values. However, the study conducted by Aun et al. [16] did not mention the mean value. The patients in most of the studies were young (20s and 30s). A study conducted by Dixon et al. [27] investigated older patients (30s to 70s) who received local anesthesia; however, there was no significant difference in the onset of effects.

Recovery from sedation

Twelve studies examined the differences in recovery periods after administration of diazepam and midazolam. Five studies reported that patients receiving diazepam took more time for recovery compared with midazolam [11,16,26,28,29], six studies reported no significant differences in recovery times [10,13,15,22,25,27], and one study reported other [21]. However, the majority of the studies reported no significant differences in recovery times for both diazepam and midazolam. Korttila and Tarkkanen [21] reported that after bronchoscopy, recovery in terms of walking and standing ability was slower in patients administered 0.1 mg/kg midazolam compared with patients administered 0.2 mg/kg diazepam. This was the only study that reported slower recovery with midazolam and, hence, was classified as "other." Therefore, the survey included 11 studies.

**Figure 5:**
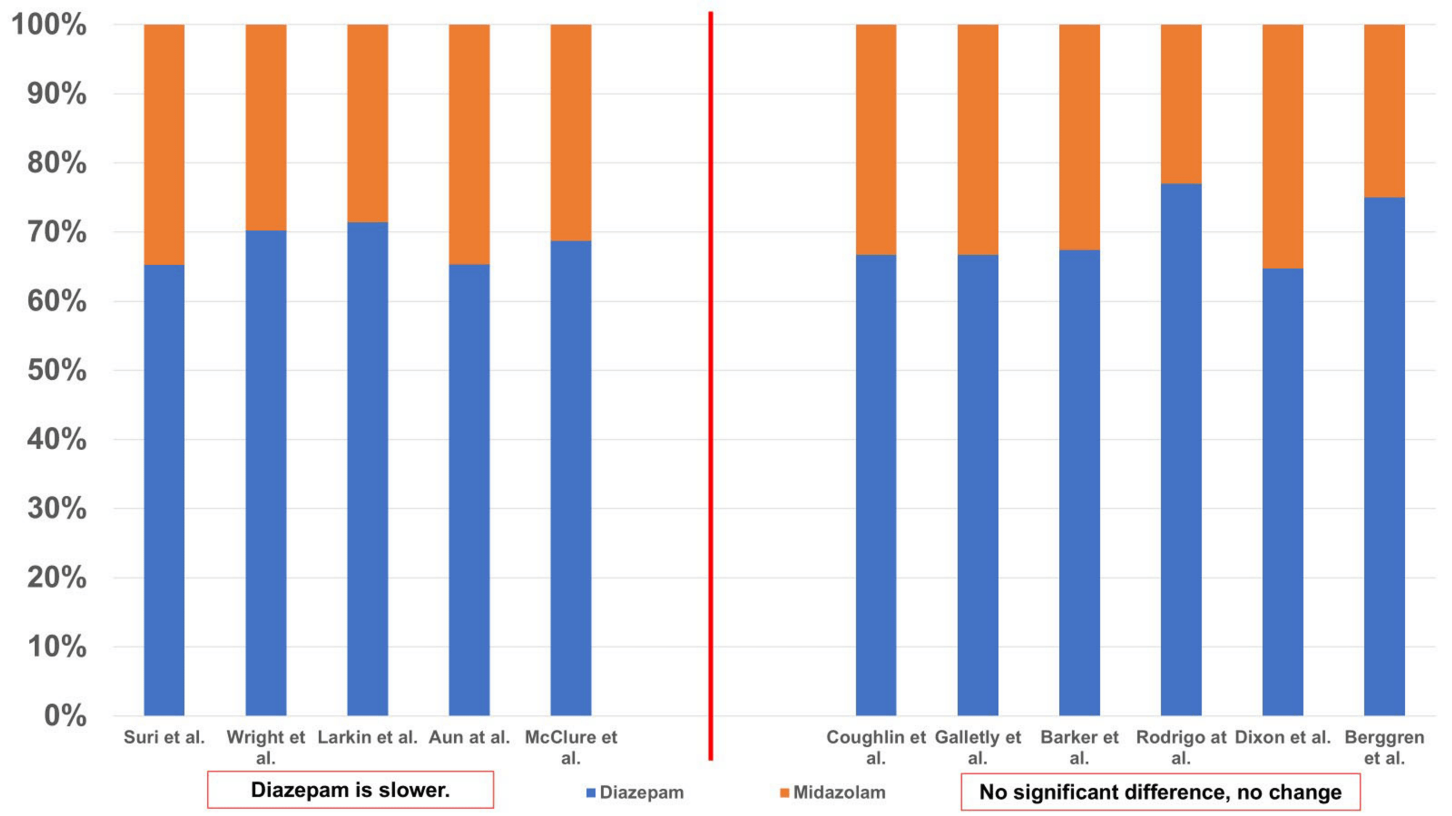
Dosage and recovery From the results of 11 studies [10,11,13,15,16,22,25-29], the age was examined. Because there was variation in the units (mg, mg/kg, mg/30 sec), they were converted to percentages. For example, 1 mg diazepam and 1 mg midazolam are 50% diazepam and 50% midazolam.

The results of 11 studies were considered to examine the dosages of diazepam and midazolam (Figure 5). Suri and Lamba [26], Wright et al. [29], Aun et al. [16], Barker et al. [13], Rodrigo and Clark [15], and Dixon et al. [27] examined the recovery of patients after administration of average doses (mg or mg/kg) of diazepam and midazolam, whereas McClure et al. [28] and Coughlin and Panuska [10] examined the recovery of patients administered the average total dose (mg or mg/kg). Additionally, Larkin and Laing [11] administered 2.5 mg/30 sec of diazepam and 1 mg/30 sec of midazolam for sedation, so these were used as the initial doses (mg). Galletly et al. [25] and Berggren et al. [22] practiced routine administration of diazepam and midazolam. The D:M ratio in studies that reported longer recovery times after diazepam than midazolam was 1.88:1 - 2.5:1, while in studies reporting no significant difference after surgery, it was 1.83:1 - 3.35:1. Thus, the dose of diazepam was reported to be higher than that of midazolam in all studies.

**Figure 6:**
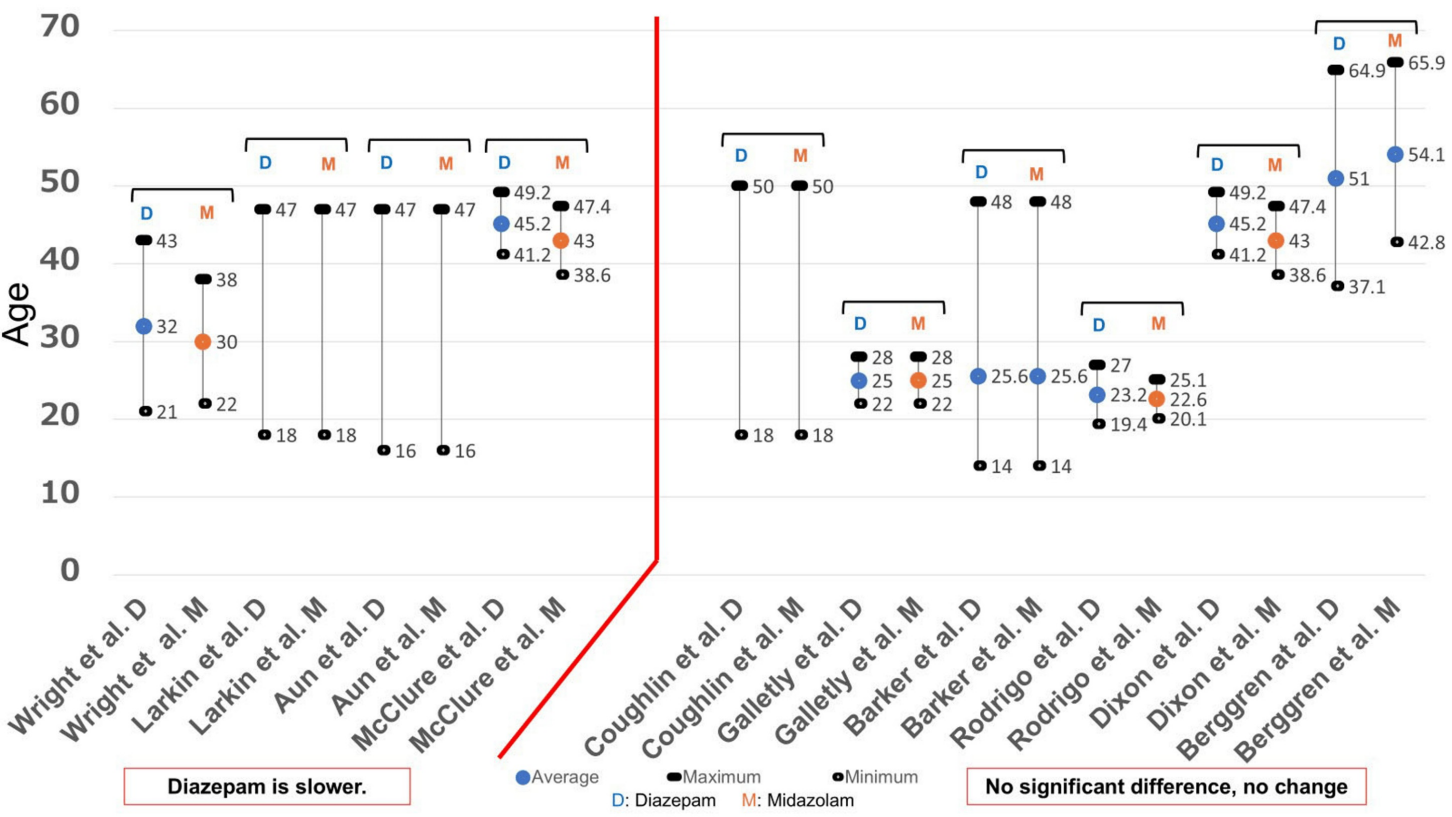
Age and recovery From the results of 11 studies [10,11,13,15,16,22,25-29], the age was examined. The maximum, minimum, and average values for each study are shown in this figure. There was one report that did not state the average, maximum, and minimum values, and three reports that did not state the average value. D: Diazepam; M: Midazolam

Since the study conducted by Suri and Lamba [26] did not include information regarding age, in this study, age was examined from the results of 10 studies (Figure 6). Almost all studies included the average, maximum, and minimum values. However, the reports by Larkin and Laing [11], Aun et al. [16], and Coughlin and Panuska [10] did not include the average value. The results of their studies included a wide range of ages, from 20 to 50 years. Berggren et al. [22] also included patients aged 51-65.9 years undergoing endoscopic examinations; however, no significant differences were found in recovery after administration.

Injection pain (vascular pain)

The difference in injection pain (vascular pain) between diazepam and midazolam was reported in 10 studies. Among these, seven studies reported that more injection pain was caused by diazepam compared with midazolam [10,11,15-17,23,29], while three studies reported that neither drug caused injection pain or that there was no significant difference in the pain caused by either drug [19,27,28]. Thus, there were more reports of diazepam causing more injection pain.

**Figure 7:**
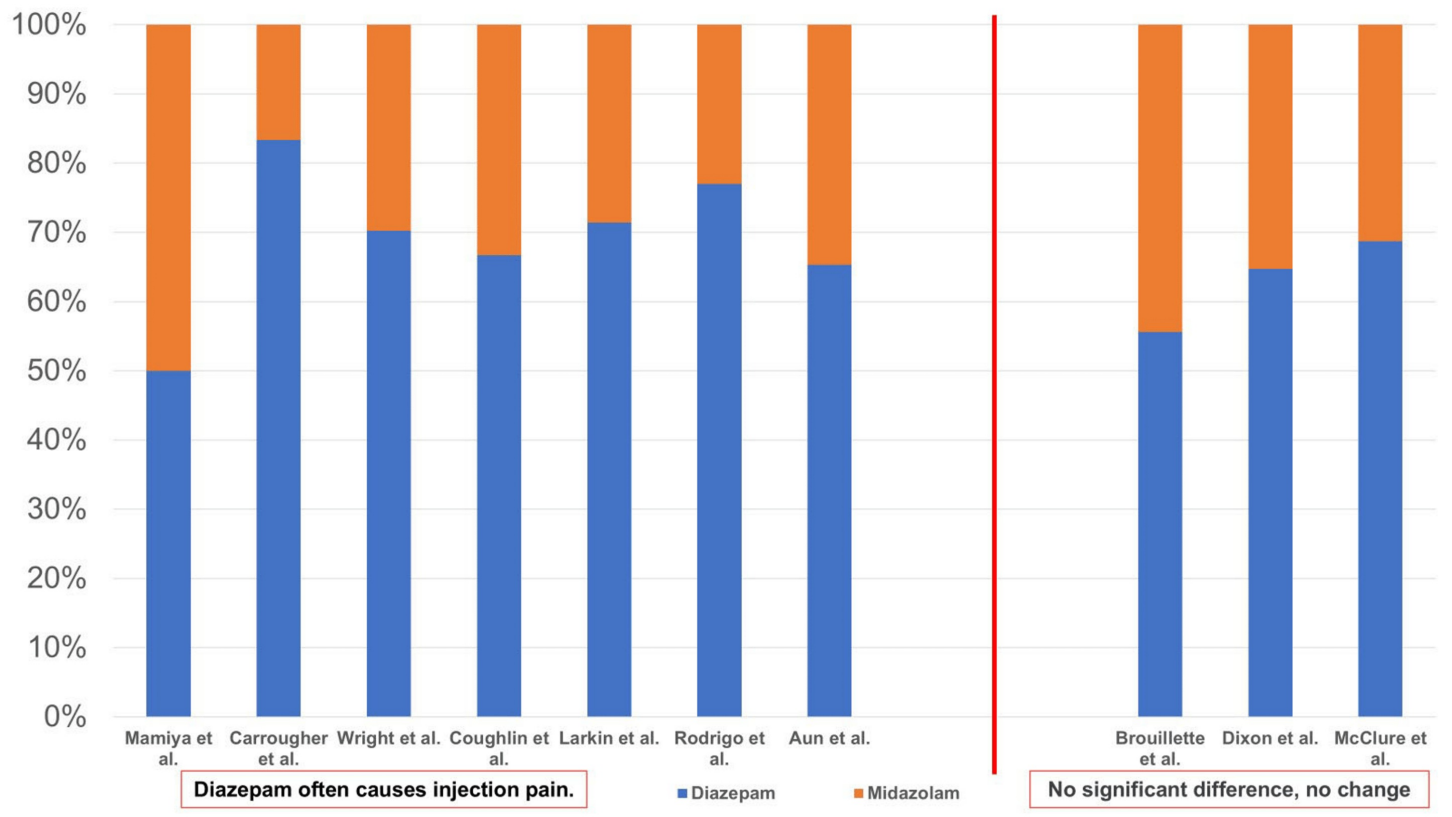
Dosage and injection pain (vascular pain) From the results of 10 studies [10,11,15-17,19,23,27-29], the age was examined. Because there was variation in the units (mg, mg/kg, mg/30 sec, mg/min), they were converted to percentages. For example, 1 mg diazepam and 1 mg midazolam are 50% diazepam and 50% midazolam.

Dosage was examined from the results of 10 studies (Figure 7). Wright et al. [29], Rodrigo and Clark [15], Aun et al. [16], and Brouillette et al. [19] examined the injection pain caused by average doses (mg or mg/kg) of diazepam and midazolam, whereas Coughlin and Panuska [10], Dixon et al. [27], and McClure et al. [28] examined the injection pain using the average total dose (mg or mg/kg). Mamiya et al. [23], Carrougher et al. [17], and Larkin and Laing [11] examined the pain caused by the amount (mg) administered over 30 sec or 1 min. The D:M ratio in reports reporting more injection pain with diazepam than with midazolam was 1:1 - 5:1, whereas the D:M ratio reporting no significant difference was 1.25:1 - 2.2:1. Thus, diazepam was found to be administered at higher doses than midazolam with the exception of the report by Mamiya et al. [23].

**Figure 8:**
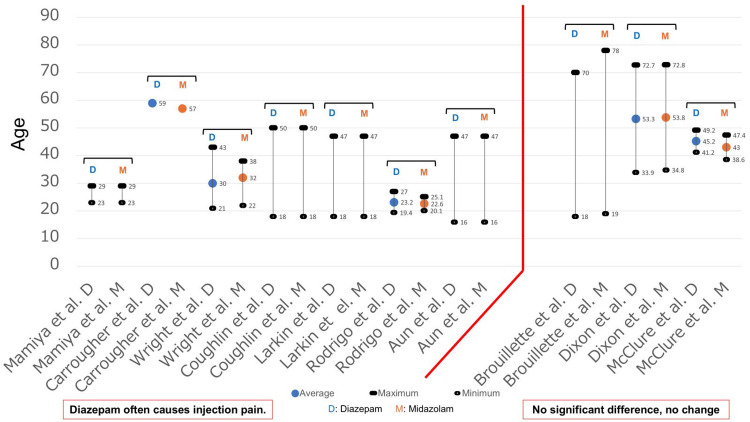
Age and injection pain (vascular pain) From the results of 10 studies [10,11,15-17,19,23,27-29], the age was examined. The maximum, minimum, and average values for each study are shown in this figure. There were five reports that did not state the average value, and one report that did not state the maximum and minimum values. D: Diazepam; M: Midazolam

Age was also examined based on the results of 10 studies (Figure 8), among which four studies [15,27-29] provided the average, maximum, and minimum values, while the remaining six studies [10,11,16,17,19,23] did not. The subjects included in studies that reported that diazepam caused more injection pain than midazolam were mainly individuals in the age group of 20-50 years, while in studies indicating no significant difference between the two, the subjects were often individuals aged 50 years and older.

Amnesic effects (anterograde amnesia)

The difference in amnesic effects (anterograde amnesia) exerted by diazepam and midazolam was investigated by 16 studies. Among these, 14 studies reported diazepam to exert a weaker amnesic effect compared with midazolam [10,11,13-16,18,20-22,25-28], and two studies reported a lack of significant differences in the amnesic effects exerted by the two drugs [12,24]. Thus, the number of reports that indicated the amnesic effects of diazepam were weaker was greater.

**Figure 9:**
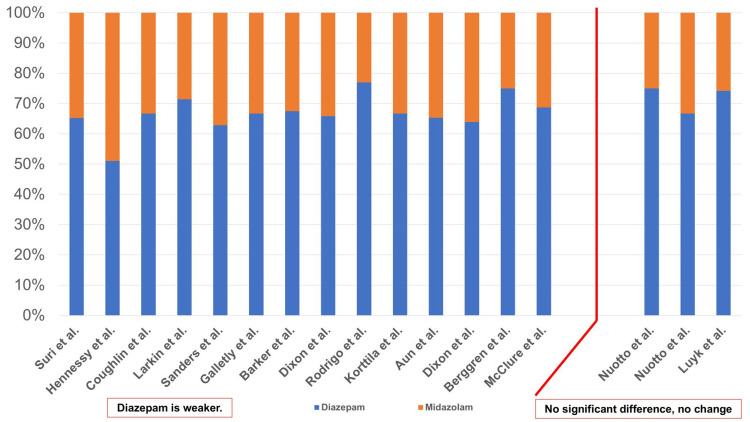
Dosage and amnesic effects From the results of 16 studies [10-16,18,20-22,24-28], the age was examined. Because there was variation in the units (mg, mg/kg, mg/30 sec, mg/min), they were converted to percentages.

With regard to the dosages of the two drugs included in the 16 studies, Suri and Lamba [26], Hennessy et al. [18], Sanders et al. [20], Barker et al. [13], Dixon et al. [14], Rodrigo and Clark [15], Aun et al. [16], and Luyk et al. [12] considered the amnesic effect exerted by the mean doses (mg or mg/kg) of diazepam and midazolam, while Coughlin and Panuska [10], Dixon et al. [27], and McClure et al. [28] considered the mean total dose (mg or mg/kg) (Figure 9). Larkin and Laing [11] considered the amount (mg) administered over 30 sec or 1 min. Galletly et al. [25] and Berggren et al. [22] routinely administered diazepam and midazolam. Korttila and Tarkkanen [21] reported that the amnesic effect exerted by midazolam 0.1 mg/kg was slightly stronger compared with diazepam 0.2 mg/kg; therefore, this dose (mg/kg) was used for the study. Nuotto et al. [24], as mentioned in Figure 4, show two results in Figure 7. The D:M ratio in the two reports ranged from 1.04:1 to 3.35:1, indicating that the amnesic effect exerted by diazepam was weaker compared with midazolam, while the D:M ratio in the reports ranged from 2:1 to 3:1, showing no significant difference. Therefore, with regard to the amnesic effect, the dose of diazepam was higher than that of midazolam in all reports.

**Figure 10:**
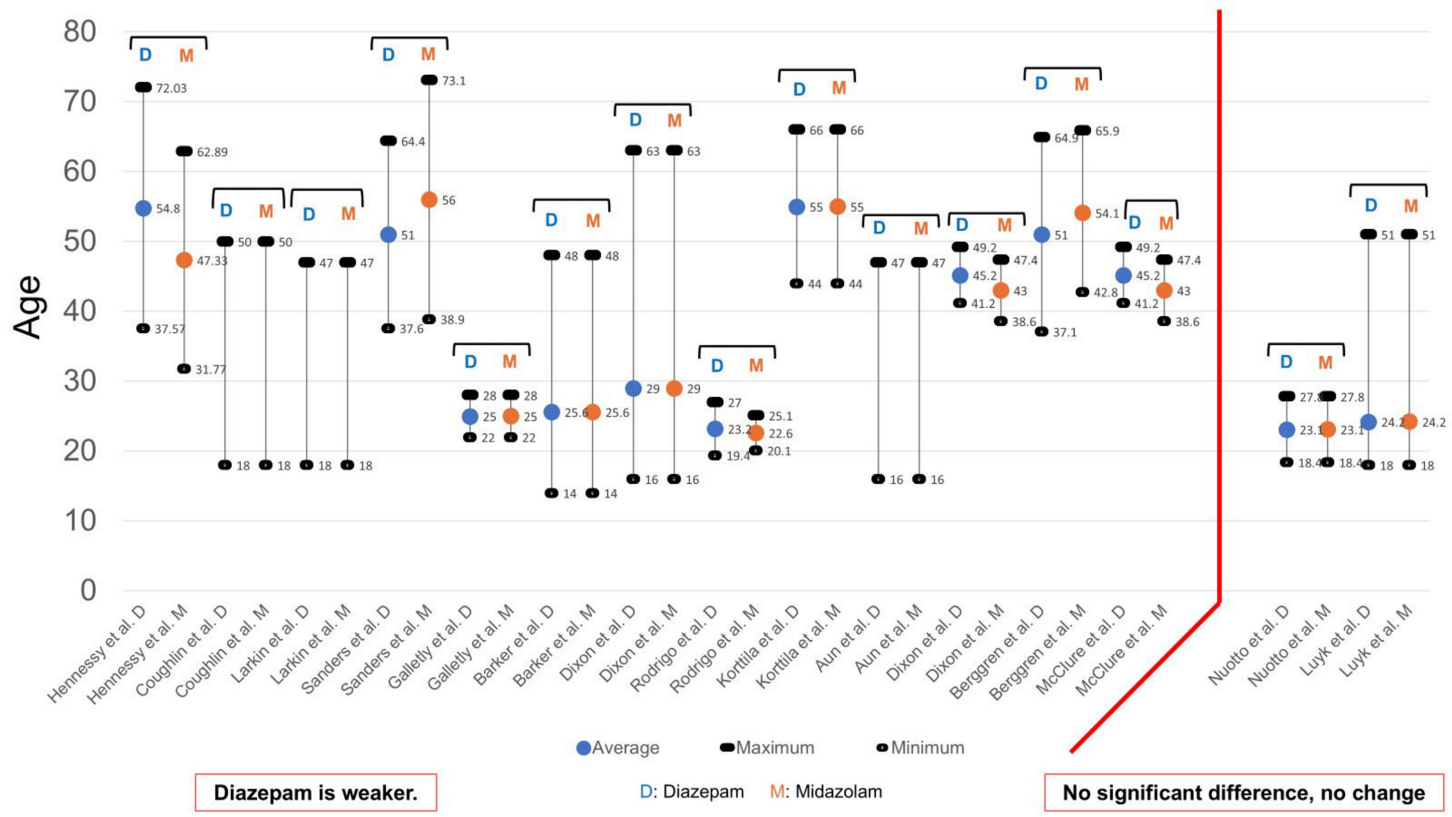
Age and amnesic effects From the results of 15 studies [10-16,18,20-22,24,25,27,28], the age was examined. The maximum, minimum, and average values for each study are shown in this figure. There were three reports that did not state the average value. D: Diazepam; M: Midazolam

Since the study conducted by Suri and Lamba [26] did not provide information regarding the ages of patients, the age of patients was examined from the results of 15 studies (Figure 10). Almost all studies provided the average, maximum, and minimum values. However, the studies conducted by Coughlin and Panuska [10], Larkin and Laing [11], and Aun et al. [16] did not provide the average values. The results indicated that diazepam exerted a weaker amnesic effect compared with midazolam and included a wide range of ages, from 14 years to 73 years; however, the reports, which demonstrated no significant difference in the amnesic effects exerted by the two drugs, included a broad age range of 18-51 years.

Discussion

In dentistry and oral surgery, IVS is practiced for patients with dental phobia, extreme gag reflex, poor cooperation, and intellectual disability [30]. In Japan, the combination of midazolam + propofol is commonly used for IVS in clinical settings [31]. The commonly practiced protocol involves the administration of an initial small dose of midazolam to achieve sedation, followed by the administration of an appropriate dose of propofol. This protects the patient from the injection pain (vascular pain) caused by propofol, due to the amnesic effect previously exerted by midazolam. Furthermore, with this combination, a reliable sedative effect can be achieved with a lower dose of propofol compared with propofol alone, along with a quicker recovery from sedation [31]. In recent years, however, there have been situations of shortages of sedatives due to infections and supply problems [2,3]. Dental anesthesiologists need viable alternatives in cases of such events.

This study aimed to evaluate the effectiveness of diazepam when midazolam is in short supply, by summarizing the literature comparing diazepam with midazolam, providing insights into the characteristics of diazepam from the perspective of modern anesthesiology, and identifying areas for future research.

According to several reports, a higher dosage of diazepam (mg) was required compared with that of midazolam in all four categories investigated in this study (onset of action, recovery from sedation, injection pain, and amnesic effects). The efficacy of midazolam, being two to seven times greater than that of diazepam (D:M ratio, ranging from 2:1 to 7:1), meant that a relatively higher dose of diazepam was required compared with midazolam, which is almost consistent with the D:M ratio observed in our study [26,32]. Unlike midazolam, diazepam has to be administered in an undiluted solution, which makes the intravenous administration of small amounts difficult, thereby making the resulting dosage higher [23]. In addition, an analysis of the results of 20 studies indicated that diazepam has a slower onset of action and recovery; it is associated with more injection pain and exerts a weaker amnesic effect compared with midazolam. Combining the findings of this review with recent knowledge in anesthesiology, a combination of diazepam and propofol could be considered for sedation in dentistry. The method of administration must be initiated by first injecting a small amount of diazepam, paying attention to the injection pain, followed by the administration of propofol to obtain optimal sedation, considering that diazepam has a slower onset of action than midazolam. Excessive diazepam can delay recovery; therefore, additional doses are avoided. Instead, sedation is maintained with propofol. This sedation method is suitable for surgeries that last for longer durations since diazepam has a longer half-life compared with midazolam. In addition, the slow onset of action of diazepam might necessitate a higher dose of propofol (mg) than that needed with the combination of midazolam and propofol. However, no case reports or clinical studies have reported on the combination of diazepam and propofol to date. Therefore, further research is needed on the optimal drug dosage for optimal sedation with diazepam and propofol, postoperative recovery time, and the presence or absence of injection pain and amnesic effects.

Most of the studies included in this review were conducted on people in the age group ranging from 20 to 50 years, and few studies were conducted on people aged 50 or older. The population of people aged 65 or older in Japan is expected to increase around 2040 [33], thereby necessitating consideration of patients who are in their 50s and older.

This study has several limitations. First, several studies that met the eligibility criteria for this study did not include information on the gender or weight of the patients, making comparisons difficult. When examining the dosage from the four categories analyzed in this study, a considerable variation was observed in the D:M ratio. There were individual differences in the dosage required to achieve optimal sedation with IVS [6]. Therefore, while gender and weight are important criteria, it would be advisable to administer diazepam while closely observing the condition of each individual patient [6].

Second, this study has targeted sedation for purposes other than dentistry. In dental and oral surgery, the surgical field and airway are in the same area, and there is a likelihood of accumulation of moisture in the oral cavity; therefore, care must be taken to prevent aspiration and airway obstruction. In addition, in recent years, advanced and lengthy dental surgeries, including implant surgery, periodontal surgery, and endodontic treatment using a microscope, have developed, and dental care is further advancing. Therefore, IVS with diazepam for these dental surgeries must also be considered.

Third, the data obtained in this study were retrieved from articles in PubMed and Google Scholar, and articles in other databases could not be examined. In addition, these articles were old, having been published several years ago, and there have been no recent clinical studies comparing diazepam and midazolam. Therefore, the results need to be interpreted with caution.

## Conclusions

In this study, the included articles were divided into four categories: onset of action, recovery from sedation, injection pain (vascular pain), and amnesic effects (anterograde amnesia). This review highlighted that diazepam (a) has a slow onset of action and recovery, (b) is more likely to cause injection pain, and (c) tends to have a weaker amnesic effect compared with midazolam, and that diazepam has to be administered at a higher dose compared to midazolam. In recent years, advances in anesthetic drugs have enabled the use of two drugs in combination. Therefore, with regard to the combination of diazepam and propofol, it is necessary to focus on the optimal drug dosage, the postoperative recovery time, and the presence or absence of injection pain and amnesic effects. Diazepam might be a favorable choice when midazolam is in short supply for IVS, especially for long dental procedures.
